# Osteonecrosis of the Femoral Head in an Adolescent on Long-Term Inhalational Corticosteroids

**DOI:** 10.1155/2017/6969787

**Published:** 2017-02-28

**Authors:** Anthony C. Egger, R. Tracy Ballock

**Affiliations:** Department of Orthopaedic Surgery, Cleveland Clinic, Cleveland, OH, USA

## Abstract

A relationship between the development of osteonecrosis of the femoral head and systemic corticosteroids has been well established in the literature, particularly in adults. However, the link between osteonecrosis and inhaled corticosteroids is less researched and understood. We report an usual case report of a 10-year-old male who developed ipsilateral femoral head osteonecrosis after long-term inhalational corticosteroid and intermittent short courses of oral steroid usage with a unique presentation and delayed diagnosis.

## 1. Introduction

Osteonecrosis is a phenomenon which results in bone cell death with subsequent disruption of bony architecture and bony collapse. The most common cause of osteonecrosis is traumatic insult, but multiple other sources have been noted. Alcohol consumption, coagulation disorders, hemoglobinopathies, and autoimmune diseases are all described, but the most frequent nontraumatic cause associated with osteonecrosis is prolonged corticosteroid usage [1]. The most common locations for osteonecrosis to occur are the femoral and humeral heads. Osteonecrosis of the femoral head most commonly affects patients in the third to fifth decades of life but can also occur in pediatric patients. The clinical presentation is often of deep groin pain, but the pain may be referred to the ipsilateral buttock or knee [1].

A link between systemic steroid use and the development of osteonecrosis of the femoral head has been well documented, but the exact mechanism of action is still debated. It has been shown that glucocorticoids can have a direct effect on bony cells through suppression of osteoblast precursor production, increasing apoptosis of osteoblasts and osteocytes, and prolonging the lifespan of osteoclasts. It has also been proposed that steroids are working through indirect means by increasing the procoagulant potential, vasoconstriction, and intraosseous pressure, all which can decrease blood flow into the femoral head [2]. Most likely steroid induced osteonecrosis results from a combination of factors with the extent of osteonecrosis determined both by the number of potential risk factors along with a patient's sensitivity and responsiveness to the steroids.

The purpose of this case report is to bring to attention femoral head osteonecrosis as a potential side effect of inhalational corticosteroid use in the pediatric population, as a delayed or missed diagnosis can lead to hip joint destruction and early arthritis.

## 2. Case Report

We present the case of a 10-year-old male presented to our institution for a second opinion regarding his recent diagnosis of Legg-Calve-Perthes disease. He initially presented to a pediatric orthopedist at an outside institution with a complaint of right knee pain without known injury. His knee pain was described as diffuse, sharp, and worse with ambulation. Initial imaging of his knee was negative. He was diagnosed with tendinitis and prescribed NSAIDs and physical therapy. He continued to have knee pain despite these modalities and was evaluated by his pediatrician who recommended crutches and nonweight bearing. An MRI was ordered but was denied by insurance. His pain continued to worsen to the point that he stopped playing football and in one occurrence prompted a visit to the emergency department. In the emergency department, imaging of his pelvis was obtained for the first time and an abnormality of the right femoral head was noted. He was referred back to the initial pediatric orthopaedic surgeon who diagnosed him with Legg-Calve-Perthes disease and advised him to continue nonweight bearing and crutches.

Initial evaluation at our institution occurred 2-3 months after symptoms began. At this stage he had started to complain of right hip pain. His past medical history was significant for asthma. He had been on daily Flovent aerosol treatment (fluticasone 110 mcg/actuation inhaler) since he was three years old. During this seven-year period he had also been on a 5-day oral prednisone regimen on six different occasions.

On physical exam he appeared in no acute distress, standing at 157.5 cm (5,2, 95% tile for age) and weighing 65.7 kg (145 lbs, 99.3%). His BMI was calculated at 26.5 which was in the 98th percentile for his age. His right hip was held in slight flexion and external rotation. There was no tenderness throughout the hip region. Hip flexion was to 90 degrees with extension to the examiner table. Hip abduction was to 40 degrees in the supine position and hip internal rotation was to 40 degrees lying prone.

**Figure 1:**
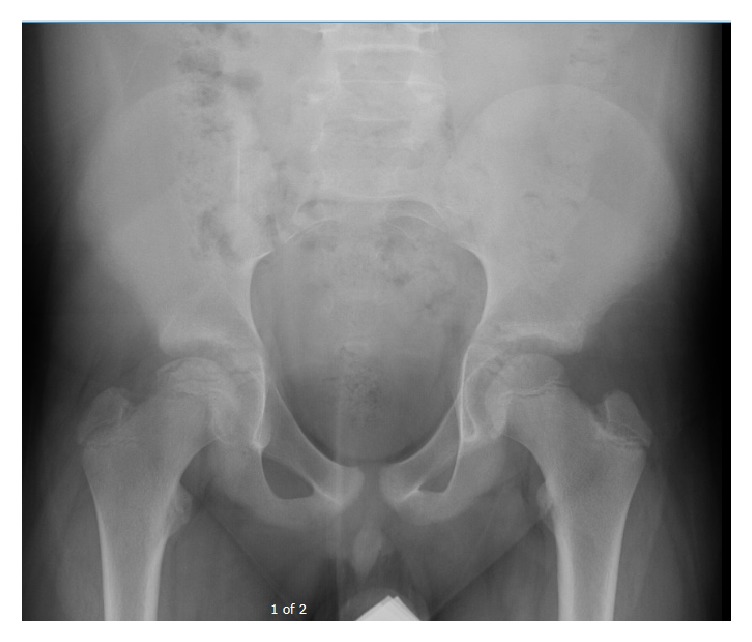
AP pelvis X-ray showing avascular changes of subchondral cystic lucency without evidence of femoral head collapse.

**Figure 2:**
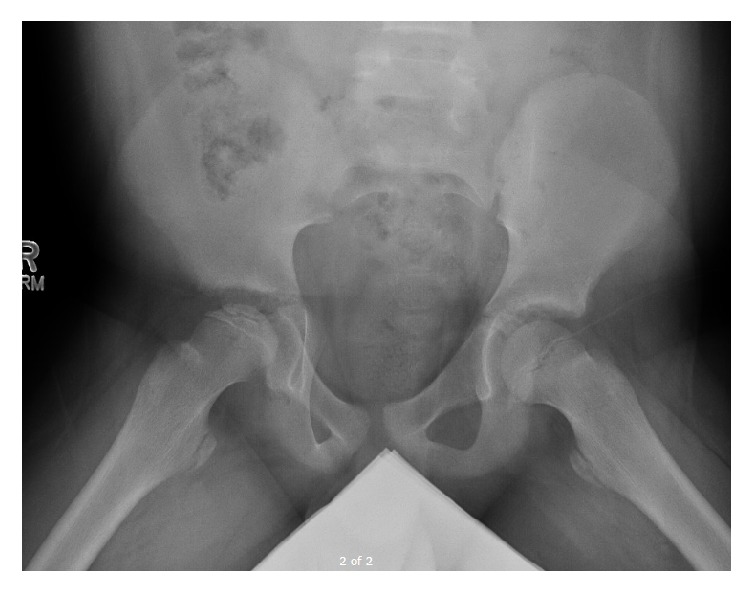
Frog leg lateral X-ray of bilateral hips again showing avascular changes with characteristic “crescent sign” of osteonecrosis to right femoral head.

Pelvic radiographs (AP and frog lateral in Figures 1 and 2) were reviewed and showed avascular changes to the femoral head with the characteristic “crescent sign” of osteonecrosis, a subchondral lucency, in the femoral epiphysis. At this time there was not yet evidence of femoral head collapse.

**Figure 3:**
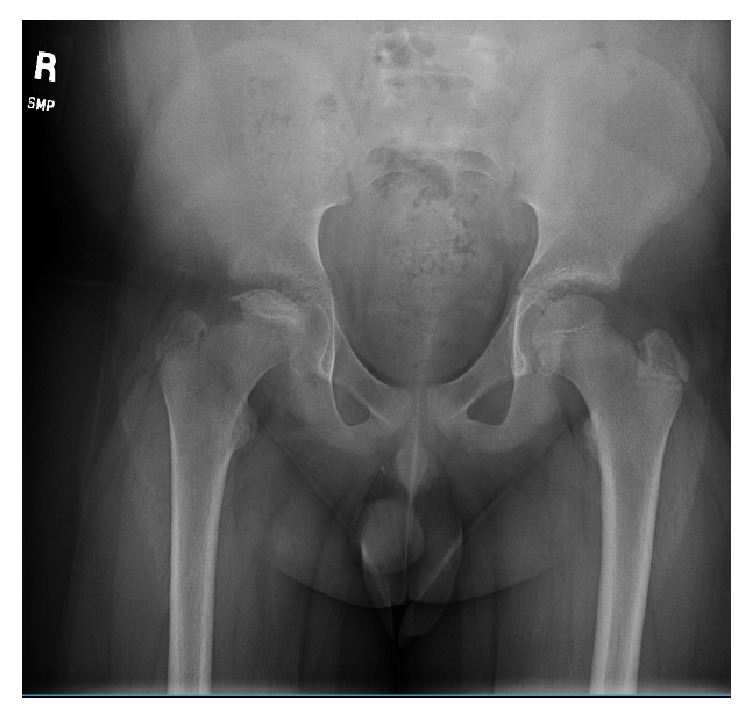
AP pelvis X-ray obtained 6 weeks from initial presentation which shows early femoral head collapse.

**Figure 4:**
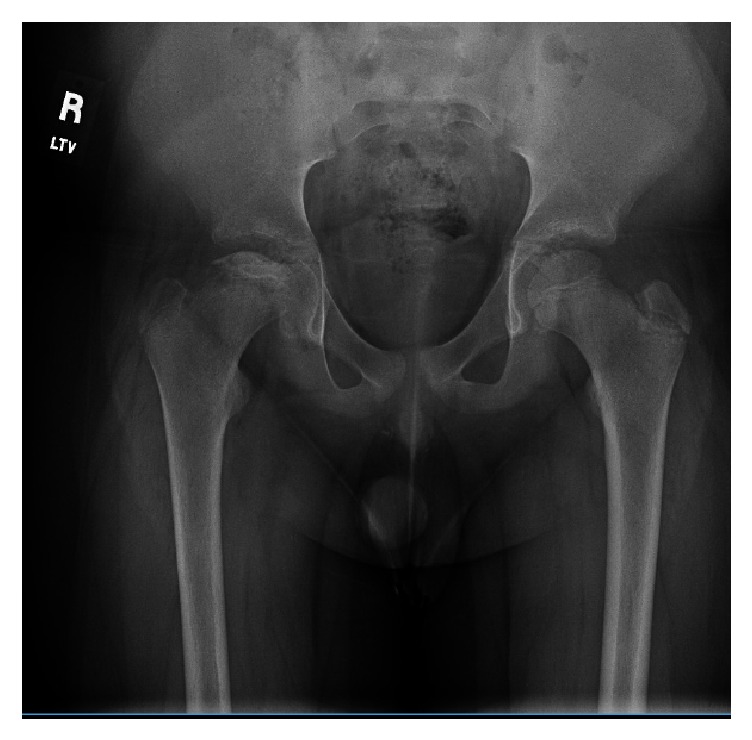
AP pelvis X-ray obtained 12 weeks from initial presentation with progressive flattening of femoral epiphysis indicative of segmental collapse seen in non-Perthes osteonecrosis.

Based on the patient's history, clinical exam, and radiographic findings, he was diagnosed with steroid induced osteonecrosis of the right hip. He was advised to continue nonweight bearing with crutches, work with physical therapy on gentle range of motion exercises, and return to clinic for repeat pelvis radiographs in six weeks. These repeat images (Figures 3 and 4) show the continued progression of the pathology, with sequential flattening of the femoral head as is expected in osteonecrosis.

## 3. Discussion

Our patient represents a unique population, as a young adolescent presenting with inhaled steroid induced femoral head osteonecrosis has not to our knowledge been previously described in the literature. The relationship between systemic steroids and osteonecrosis has been established, particularly in regard to adults and systemic steroids [3]. The information on the role of inhaled corticosteroids is markedly less publicized, but their use has been shown to have a negative effect on bone health, especially in regard to impeding bone remodeling [4]. Fluticasone propionate, one of the most prescribed medications for chronic asthma, has in particularly been shown to exhibit greater dose related systemic bioactivity compared to other available inhaled corticosteroids, especially at doses >0.8 mg/d [5]. The Flovent HFA® (Glasgow Smith Klein) full product report does note that inhaled corticosteroids are a risk for development of osteonecrosis, as well as a potential for reduction in growth velocity and bone mineral density in children.

The World Health Organization has also recognized the potential risk and published an advisory warning based on five-case reports of osteonecrosis thought to be associated with inhaled fluticasone propionate [6]. These cases were all in adult subjects, and no cases were associated with the use of topical or inhaled steroids alone. Karkoulias et al. describe a case report of a 38-year-old male who developed bilateral femoral head osteonecrosis without any metabolic, coagulopathic, or lifestyle related risk factors but a long-term history of inhaled fluticasone propionate, intranasal triamcinolone acetonide, and intermittent oral corticosteroids for chronic asthma [7]. Kaviani et al. describe another case of bilateral hip osteonecrosis in a patient receiving inhaled corticosteroids, but this patient also was infected with HIV and on antiretroviral therapy [8].

In the pediatric literature, a case report from Italy describes a 2-year-old girl who developed bilateral femoral head osteonecrosis associated with recurrent myopathy and bone symptoms after a single short-term oral treatment and three courses of inhaled corticosteroids for wheezing. Other than the corticosteroid use, she had no known risk factors for the development of osteonecrosis. However, upon further testing she was found to have an increased number of glucocorticoid receptor sites per cell and thus an increased sensitivity to the medication [9].

As with our patient, all prior case reports documenting osteonecrosis potentially related to inhalational osteonecrosis were complicated by the patient utilization of oral steroids at some time point, but none were described in this age group. This is to be expected, given that those patients suffering from asthma severe enough to be on long-term inhalational steroids would likely at some point in the course of their disease require systemic dosing for an acute flare. Though it is difficult to determine direct causality in the development of our patient's femoral head osteonecrosis, given no other known risk factors, it is suspected that the large cumulative dose of inhalational steroids received over 7 years of treatment in addition to the intermittent oral courses were associated with the development and progression of the disease.

There is a possibility that this case could be an abnormal manifestation of Legg-Calve-Perthes disease. There are documented cases of Perthes in young adolescents, but the majority of Perthes cases occur in ages 4–8. Also in Perthes, the hip progresses through radiographic stage of epiphyseal fragmentation and subsequent reossification rather than segmental collapse as has occurred with our patient. Obesity has also been noted as a potential source of idiopathic osteonecrosis. Zhao et al. noted obesity as a risk factor in a large review of Chinese patients aged 15 years or older [10]. They also note a relationship with hyperlipidemia as has been shown before [11]. However, both of these studies evaluated a more adult population and there has been no evidence to note the relationship of obesity to idiopathic osteonecrosis in the young adolescent population. Thus, given the patient's age on presentation, radiographic progression, and the evidence in the literature of a relationship between osteonecrosis and inhalational and oral steroid use, it is our belief that inhaled steroids are the most likely cause of this patient's hip osteonecrosis.

Given the large number of inhalers prescribed for asthma yearly and the long-term consequences of femoral head osteonecrosis, it is important for healthcare providers to be aware of this rare but important potential complication of inhaled steroid use. More research is needed to further delineate the relationship between inhaled steroids and osteonecrosis, but those younger patients on long-term inhaled corticosteroids who complain of knee or hip pain should be evaluated for potential osteonecrosis. It is particularly important for the primary care providers who prescribe these medications to be aware of this potential association, counsel patients and parents accordingly, and prompt timely investigation when needed as the treatment options for advanced disease are often limited and when missed can lead to children with painful, arthritic hips at an early age.
